# Improvement of Insulin Sensitivity by Isoenergy High Carbohydrate Traditional Asian Diet: A Randomized Controlled Pilot Feasibility Study

**DOI:** 10.1371/journal.pone.0106851

**Published:** 2014-09-16

**Authors:** William C. Hsu, Ka Hei Karen Lau, Motonobu Matsumoto, Dalia Moghazy, Hillary Keenan, George L. King

**Affiliations:** 1 Asian American Diabetes Initiative, Joslin Diabetes Center, Harvard Medical School, Boston, Massachusetts, United States of America; 2 Dianne Nunnally Hoppes Laboratory for Diabetes Complications, Joslin Diabetes Center, Harvard Medical School, Boston, Massachusetts, United States of America; 3 R&D Department, Sunstar Inc., Osaka, Japan; San Raffaele Hospital, Italy

## Abstract

The prevalence of diabetes is rising dramatically among Asians, with increased consumption of the typical Western diet as one possible cause. We explored the metabolic responses in East Asian Americans (AA) and Caucasian Americans (CA) when transitioning from a traditional Asian diet (TAD) to a typical Western diet (TWD), which has not been reported before. This 16-week randomized control pilot feasibility study, included 28AA and 22CA who were at risk of developing type 2 diabetes. Eight weeks of TAD were provided to all participants, followed by 8 weeks of isoenergy TWD (intervention) or TAD (control). Anthropometric measures, lipid profile, insulin resistance and inflammatory markers were assessed. While on TAD, both AA and CA improved in insulin AUC (−960.2 µU/mL×h, P = 0.001) and reduced in weight (−1.6 kg; P<0.001), body fat (−1.7%, P<0.001) and trunk fat (−2.2%, P<0.001). Comparing changes from TAD to TWD, AA had a smaller weight gain (−1.8 to 0.3 kg, P<0.001) than CA (−1.4 to 0.9 kg, P = 0.001), but a greater increase in insulin AUC (AA: −1402.4 to 606.2 µU/mL×h, P = 0.015 vs CA: −466.0 to 223.5 µU/mL×h, P = 0.034) and homeostatic static model assessment-insulin resistance (HOMA-IR) (AA: −0.3 to 0.2, P = 0.042 vs CA: −0.1 to 0.0, P = 0.221). Despite efforts to maintain isoenergy state and consumption of similar energy, TAD induced weight loss and improved insulin sensitivity in both groups, while TWD worsened the metabolic profile.

*Trial Registration:* ClinicalTrials.gov NCT00379548

## Introduction

Over the last decade, the prevalence of diabetes has drastically increased in Asia. In 2011, over 50% of the world's diabetes cases amongst adults were from South East Asia and the Western Pacific [1]. Diabetes prevalence increased from 2.5% in 1994 to 9.7% recently in China, with similar trends observed in Japan, India and Vietnam. Further, the prevalence of diabetes is higher in urban areas than rural regions in Asia by 2–3 fold, correlating to their exposure to Western culture [2].

The progression of the diabetes epidemic among Asian Americans (AA) reflects a similar trend as reported in the New York City Health and Nutrition Examination Survey conducted in 2004. Compared to non-Hispanic white, black and Hispanic subjects, Asians have the highest prevalence of diabetes and impaired fasting glucose (IFG) [2], [3].

The phenotype of diabetes in Asians differs from other ethnicities. Asians predominately have type 2 diabetes, with significantly lower rates of type 1 diabetes than Caucasians. Type 2 diabetes in Asians occurs at a lower body mass index (BMI) of 24–25 kg/m^2^ as reported in several large studies [2], [4], [5], and has suggested a lower BMI cutoffs for overweight and obese [6], [7]. Although genetics may contribute to the differences, environmental factors such as diet and the transition to sedentary lifestyles also contribute to the increased development of diabetes [8].

Change in diet is likely one of the environmental factors contributing to the epidemic of type 2 diabetes in Asians and AA [8]. A close examination of the macronutrient distribution of a traditional Asian diet (TAD) and a typical Western diet (TWD) showed many differences. Similar to other rural diets [9]–[12], TAD is high in carbohydrates (55–70% total energy intake) and fiber (approximately 33 g/per day), while low in fat (15% total energy intake) and animal-based protein (20% protein intake) [12]. In contrast, TWD is lower in carbohydrates (50% total energy intake) and fiber (10–12 g/per day), while higher in fat (34% total energy intake). The amount of protein composition is similar between TAD (15%) and TWD (16%), but TWD contains protein mainly of animal origin (60–80% protein intake) [13].

Many studies have characterized the effects of TWD on physiological and inflammatory responses in humans [14]–[19], which have been associated with increased insulin resistance and diabetes. Diets similar to TAD have been reported to reduce risks of cardiovascular disease, although the effects of its high carbohydrate content on the physiology of people at risk for diabetes have not been characterized. In addition, no studies have compared the metabolic responses to TAD and the transition to TWD in AA and Caucasian Americans (CA). Thus, we have designed a randomized control pilot and feasibility study to compare the effects of TAD and TWD on metabolic risk factors for diabetes in both AA and CA subjects who are at risk for type 2 diabetes.

## Research Design and Methods

### Study design

This 16-week randomized control pilot feasibility study was designed to characterize the metabolic impact of changing from TAD to TWD in AA and CA who are at higher risk of developing T2DM. After 2 weeks of run-in period which participants followed their habitual dietary intake, participants were randomized into the control group (16 weeks of TAD), or the intervention group (8 weeks of TAD, followed by 8 weeks of TWD).

This study measured the primary outcome of glycemic metabolic parameters, and secondary outcomes of anthropometric measurements, inflammatory markers, oxidative factors, lipid profile, adiposity and blood flow. General health perspective, diet palatability and dietary adherence were also assessed.

### Participants

This pilot feasibility study included 50 participants of CA and East Asian Americans (Chinese, Japanese or Korean) who shared similar macronutrient distribution in their diet. Participants aged 25–55 and at risk for diabetes were enrolled and conducted follow-ups from January 4, 2006 to June 30, 2008. Random number assignment was used for randomization; a random number list was generated by the consulting biostatistician using SAS and assigned chronologically to each participant after enrolling into the study according to ethnic group. With an even distribution of participants in each ethnic group, five from each group were randomized into the control.

All participants had either a family history (first or second degree) of type 2 diabetes, and/or a medical history of gestational diabetes, IFG or impaired glucose tolerance (IGT). Subjects were required to be within healthy weight and overweight range (BMI of 18.5–27 kg/m^2^, in accordance with the Asian Americans BMI cutoff) [6], [7] and agreed to maintain their habitual physical activity throughout the study. Most participants were recruited from family members of patients at a tertiary diabetes clinic who had agreed to be contacted for potential research studies. Participants were also recruited through newspaper advertisements, flyers, or by referrals from colleagues. Smokers and individuals who had diabetes, drug or alcohol abuse, pregnancy, medical history of vascular disease, autoimmune or inflammatory conditions, acute weight change over the past 6 months (±1.8 kg or more per month), and anyone who did not agree to comply with the study diets, and those who were on medications such as statins, angiotensin converting enzyme inhibitor, warfarin, or any other anti-inflammatory medications were excluded. Recruitment was ended when the enrolment reached 50 participants.

This study was approved by the Scientific Advisory Committee at Beth Israel Deaconess Medical Center, Institutional Review Board at Harvard Dental School and Joslin Diabetes Center in Boston, Massachusetts. All participants provided written informed consent. The protocol for this trial and supporting CONSORT checklist are available as supporting information; see Checklist S1 and Protocol S1.

### Screening

Pre-screening in concordance with inclusion/exclusion criteria was conducted via phone. Qualified individuals were invited to the clinical research center to complete the first visit. Fasting blood test and 75 g oral glucose tolerance test (OGTT) were conducted to exclude those with blood glucose (BG) ≥200 mg/dL at 2-hour post-glucose-load or ≥126 mg/dL at fasting. Physical examinations, blood pressure, pregnancy tests, and anthropometric measurements were performed. A Registered Dietitian (RD) conducted a 24-hour diet recall and counseled all participants regarding diet compliance and habitual physical activity maintenance. Qualified individuals returned for three more visits during the study.

### Diet

A RD in consultation with professional culinary staff designed recipes with a 10-day menu cycle that reflected the nutrition composition of TAD and TWD. TAD consisted of 70% energy from carbohydrate, 15% from protein, 15% from fat, and 15 g fiber/1,000 kcal [20]; TWD contained 50% energy from carbohydrate, 16% from protein, 34% from fat, and 6 g fiber/1,000 kcal [13]. The nutrient content of the diets were evaluated by nutritional software (Pronutra version 3.4.0.0; Viocare, Princeton, NJ). Other Asian food items not in the database were added using food nutrient references from Japan (Standard Tables of Food Composition in Japan Fifth Revised and Enlarged Edition 2006).

The amount of food provided to each participant was determined by their daily energy need. It was calculated using the Mifflin-St. Jeor Equation based on age, height, weight, gender and activity level that was determined by International Physical Activity Questionnaire (IPAQ) 7-day short form [20]–[23]. To ensure the outcomes were not confounded by the effects of weight change, all participants were weighed every two weeks; the amount of food provided were adjusted as necessary by the RD to ensure that participants' weight was maintained within ±2 kg from their weight at the start of the study.

The RD trained the professional culinary staff to prepare the recipes including properly measuring and weighing the food to ensure standardized measurement and food preparation. A delivery company was contracted to deliver the food to participants every 2-3 days for freshness and quality. Three meals and one snack were included each day. Daily weighed food records were provided to each participant. Participants were asked to record the percentage of consumption of the food provided daily. Participants were instructed to consume only foods provided through the study, and limit eating out, drinking alcohol or eating high fat meals. Participants were asked to record all the non-study provided food that they consumed during the 16-week study.

All participants took a multivitamin daily during the study to minimize the potential effects of the differences in micronutrients on metabolism and health.

### Study flow

**Figure 1:**
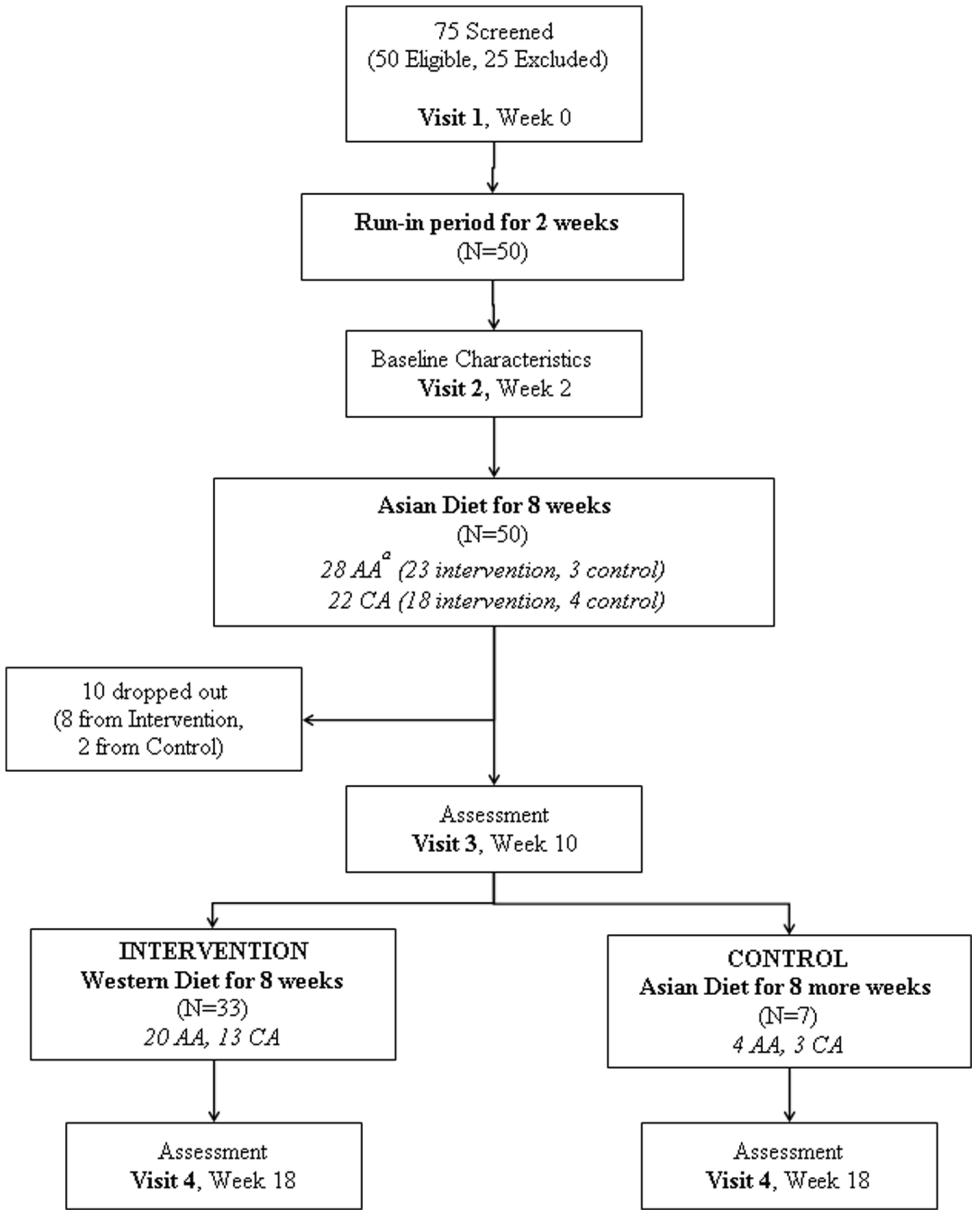
Flow of participants throughout the study. ^a^AA, Asian Americans; CA, Caucasian Americans.

There were four study visits – screening, before the first 8 weeks of TAD, after the first 8 weeks of TAD and after the last 8 weeks of TAD or TWD. At each visit, participants arrived at the clinical research center after fasting overnight for 10 hours. Blood tests, 75 g OGTT, urine albumin test, anthropometric measurements, vital signs, dual-emission X-ray absorptiometry (DXA) scans and forearm Doppler Sonography were performed. Subjects were also asked to complete questionnaires, including the IPAQ 7-day short form, general health survey and palatability survey (Fig. 1).

### Statistical analysis

Descriptive statistics were done on the *a priori* variables of interest including glycemic metabolic parameters, anthropometric measurements, inflammatory markers, oxidative factors, lipid profiles, brachial artery flow and adiposity markers. The changes in these variables were compared using the Kruskal-Wallis and Chi-square tests with a type 1 error of 0.05 between the two ethnic groups to determine if there were significant differences at baseline. The change in the value over the exposure period was calculated by subtracting the value of the latter visit from the former (e.g. visit 3-visit 2). The changes over the two exposure periods were compared between the intervention and control group, and between the ethnic groups using two-way non-parametric comparison under an intent-to-treat model. The predictive effect of weight change on body fat, trunk fat, BG AUC, insulin AUC and HOMA-insulin resistance (HOMA-IR) were performed using regression analysis.

## Results

Seventy-five individuals were screened and 50 subjects (28 AA, 22 CA) met participation criteria. Forty participants (24 AA, 16 CA) completed the study; 9 of them were randomized into the control group, while 41 of them were in the intervention group. Two from the control group (1 AA, 1 CA), and 8 from the intervention group (3 AA, 5 CA) discontinued during the first 8 weeks of the study. Of these, three left due to issues regarding food preferences, three due to restriction on social activities, three for non-compliance with the study diet and one due to pregnancy. Seven in the control group and 33 in the intervention group completed the study.

Participants (N = 50) had an average age of 34.4±8.4 and BMI of 23.3±2.6 kg/m^2^. Among the 28 AA, nine (32.1%) had IFG or IGT, while three (13.7%) of the 22 CA had IGT, and none had IFG. At baseline, age and gender, BMI and waist-to-hip ratio were not significantly different in either group (Table 1). Despite having similar mean HbA_1c_ (P = 0.179), BMI (P = 0.241) and HOMA-IR (P = 0.309) between AA and CA, AA had significantly higher insulin AUC (P = 0.015) than CA. As for inflammatory markers, AA had significantly lower adiponectin (P = 0.046), and significantly higher plasminogen activator inhibitor-1 (PAI-1)(P = 0.007) and interleukin (IL)-8 (P = 0.006) than CA. In contrast and interestingly, AA had a lower C-reactive protein (CRP) than CA with a borderline significance (P = 0.055).

**Table 1:** Baseline Profile for all participants enrolled Asian Americans vs Caucasian Americans (N = 50).

	Asian Americans (n = 28)	Caucasian Americans (n = 22)	P-value
**Age**	34.9±8.7	33.7±8.2	0.550
**Gender**			
** Male**	7 (14.0% of all participants)	9 (18.0% of all participants)	
** Female**	21 (42.0% of all participants)	13 (26.0% of all participants)	
**IFG** a	3 (10.7% of all AA)	0 (0% of all CA)	
**IGT**	6 (21.4% of all AA)	3 (13.6% of all CA)	
**Height (cm)**	163.1±9.7	171.1±7.7	0.004
**Weight (kg)**	61.2±11.5	69.6±9.5	0.010
**BMI (kg/m^2^)**	22.9±2.8	23.7±2.3	0.241
**HbA_1c_ (%)**	5.4±1.1	5.4±0.3	0.179
**Body Fat %**	26.5±5.3	27.5±7.5	0.632
**Trunk Fat %**	25.7±6.4	25.2±7.4	0.799
**Waist-to-hip ratio**	0.9±0.1	0.9±0.1	0.660
**BP systolic (mmHg)**	109.9±11.9	112.0±9.7	0.595
**BP diastolic (mmHg)**	71.0±9.8	69.6±5.8	0.979
**Insulin AUC (µU/mL × h)**	5758.2±3782.7	3557.8±1291.3	0.015
**Glucose AUC (mg/dL × h)**	15973.0±3526.5	13252.9 ± 3438.5	0.012
**HOMA-IR**	1.4±1.1	1.1±0.5	0.309
**Total Cholesterol (mg/dL)**	182.9±35.5	176.7±40.2	0.469
**Triglycerides (mg/dL)**	102.1±113.0	75.3±64.0	0.082
**HDL (mg/dL)**	53.7±17.3	58.4±16.7	0.314
**LDL (mg/dL)**	110.6±35.7	99.3±27.3	0.207
**Microalbumin (µg albumin/mg creatinine)**	10.4±12.6	10.9±10.8	0.953
**%B**	6.3±2.2	6.2±3.8	0.389
**%N**	19.8±5.4	19.4±9.1	0.591
**CRP (ng/mL)**	774.9±864.4	2601.2±3169.3	0.055
**Leptin (ng/mL)**	9.2±4.9	12.4±9.1	0.412
**Adiponectin (ng/mL)**	14339.9±9830.0	19128.2±8340.7	0.046
**PAI-1 (ng/mL)**	72.8±179.2	23.9±14.6	0.007
**IL-1B (serum)(pg/mL)**	10.4±5.5	8.5±4.9	0.214
**IL-6 (serum)(pg/mL)**	3.3±2.2	3.0±1.8	0.675
**TNF-α (pg/mL)**	8.5±3.9	9.3±3.5	0.230
**IL-8 (pg/mL)**	18.5±6.1	12.7±7.3	0.006
**Isoprostane (ng/mL)**	1.9±1.3	2.6±2.4	0.571

a BP, blood pressure; CRP, C-reactive protein; HbA_1c_, glycosylated hemoglobin; HOMA-IR, Homeostatic model assessment-insulin resistance; IFG, impaired fasting glucose; IGT, impaired glucose tolerance; OGTT, oral glucose tolerance test; PAI-1, plasminogen activator inhibitor-1; % B, endothelium-mediated dilation; % N, non-endothelium-mediated dilation; IL, interleukin; TNF-α, tumor necrosis factor-alpha.

For both intervention and control groups, 90% of the food provided was consumed. During TAD, weight change of −1.9±1.5 kg (P<0.001) was observed for all study completers, even with intense biweekly energy adjustments with RD on meal plans to avoid weight loss. In contrast, when study subjects were on TWD (N = 33), weight gain of 0.7±1.3 kg (P = 0.009) was observed. For the intervention group during TWD, the energy intake was 39.1±113.9 kcal/day higher than TAD (P = 0.048). As for the control group (N = 7), participants consumed an insignificant extra 119.8±175.6 kcal/day (P = 0.128) during the second 8 weeks of TAD than the first 8 weeks of TAD, and was associated with a weight loss of −1.5±1.5 kg (P = 0.028) during the first 8 weeks and continued to have an insignificant weight loss during the second 8 weeks of TAD −0.4±1.9 kg (P = 0.866).

Based on the IPAQ 7-day short form, no significant changes in activity levels were found in AA and CA in both control and intervention groups throughout the study.

Using intent-to-treat analysis, Table 2 shows the physiological changes of all subjects who were in the intervention group (N = 41). Weight (P<0.001), BMI (P<0.001), body fat (P<0.001) and trunk fat (P<0.001) were significantly reduced during TAD then increased during TWD. Insulin AUC was significantly reduced after TAD (P = 0.001). Total cholesterol (P<0.001), HDL (P<0.001) and LDL (P<0.001) were significantly decreased during TAD while increased during TWD, but the HDL:LDL ratio was not changed significantly. Leptin (P<0.001) and adiponectin (P = 0.020) were significantly reduced during TAD but significantly increased on TWD.

**Table 2:** Physiological Responses to Different Diets in Intervention Group, including drop-outs (Intent-to-treat) (N = 41).

	TAD^a^	P-value^b^	TWD	P-value^c^	P-value^d^
	ΔVisit 3-Visit 2		ΔVisit 4-Visit 3		
**Weight (kg)**	−1.6±1.5	<0.001	0.5±1.2	0.009	<0.001
**BMI (kg/m^2^)**	−0.6±0.6	<0.001	0.2±0.5	0.048	<0.001
**Body Fat %**	−1.7±1.6	<0.001	0.9±1.5	<0.001	<0.001
**Trunk Fat %**	−2.2±1.9	<0.001	1.1±2.0	0.001	<0.001
**Waist-to-hip ratio**	0.0±0.0	0.801	0.0±0.0	0.172	0.465
**BP systolic (mmHg)**	1.6±13.2	0.557	−3.2±7.6	0.023	0.182
**BP diastolic (mmHg)**	−0.2±9.1	0.716	0.1±8.1	0.990	0.907
**Insulin AUC (µU/mL × h)**	−960.2±1775.5	0.001	438.2±1590.4	0.208	0.001
**Glucose AUC (mg/dL × h)**	−1014.2±2098.4	0.011	−161.7±2093.2	0.611	0.110
**HOMA-IR**	−0.3±0.7	0.022	0.1±0.6	0.339	0.018
**Total Cholesterol (mg/dL)**	−25.6±23.9	<0.001	20.4±21.1	<0.001	<0.001
**Triglycerides (mg/dL)**	9.5±48.4	0.526	13.0±94.8	0.674	0.852
**HDL (mg/dL)**	−10.4±9.6	<0.001	8.2±8.8	<0.001	<0.001
**LDL (mg/dL)**	−14.4±18.8	<0.001	8.1± 14.7	0.001	<0.001
**Microalbumin (µg albumin/mg creatinine)**	−1.9±7.7	0.067	0.0±10.2	0.286	0.075
**%B**	1.1±2.9	0.028	−0.5±2.3	0.150	0.057
**%N**	−1.5±3.6	0.009	0.9±3.6	0.080	0.011
**CRP (ng/mL)**	−270.0±2160.7	0.343	−190.9±1684.9	0.427	0.533
**Leptin (ng/mL)**	−2.6±4.0	<0.001	2.1±4.7	0.001	<0.001
**Adiponectin (ng/mL)**	−1748.2±5626.0	0.074	2877.6±8381.0	0.046	0.020
**PAI-1 (ng/mL)**	−8.4±23.4	0.003	−3.2±40.3	0.020	0.002
**IL-1B (serum)(pg/mL)**	2.5±6.4	0.037	4.6±6.3	<0.001	0.339
**IL-6 (serum)(pg/mL)**	0.2±1.9	0.480	−0.1±1.8	0.772	0.623
**TNF-α(pg/mL)**	0.1±5.5	0.851	0.0±4.2	0.900	0.796
**IL-8 (pg/mL)**	1.0±7.0	0.688	−1.7±5.6	0.112	0.304
**Isoprostane (ng/mL)**	−0.2±1.6	0.765	0.8±1.7	0.002	0.048

a BP, blood pressure; CRP, C-reactive protein; HOMA-IR, Homeostatic model assessment-insulin resistance; OGTT, oral glucose tolerance test; PAI-1, plasminogen activator inhibitor-1; % B, endothelium-mediated dilation; % N, non-endothelium-mediated dilation; IL, interleukin; TAD, traditional Asian diet; TNF-α, tumor necrosis factor-alpha; TWD, typical Western diet.

b P-value of the changes observed before and after 8 weeks of TAD (Visit 3-Visit 2).

c P-value of the changes observed before and after 8 weeks of TWD (Visit 4-Visit 3).

d P-value of the Visit 4-Visit 3 and Visit 3-Visit 2.

Table 3 shows the specific physiologic responses of AA (N = 23) and CA (N = 18) in the intervention group after TAD and TWD, using intent-to-treat analysis. Despite biweekly energy adjustments to maintain isoenergy state throughout the study, both AA and CA experienced 3% weight loss (AA: P<0.001; CA: P = 0.001). The body fat (AA: P<0.001; CA: P = 0.001) and trunk fat (AA: P<0.001; CA: P = 0.001) decreased after TAD while increased after TWD. Insulin AUC was significantly reduced after TAD while increased after TWD for both AA (P = 0.015) and CA (P = 0.034), but only AA had a significant change in HOMA-IR (P = 0.042). Decrease in glucose AUC was only observed among AA after TAD (P = 0.025). Total cholesterol (AA: P = 0.001; CA: P = 0.001), HDL (AA: P<0.001; CA: P = 0.001) and LDL (AA: P = 0.014; CA: P = 0.001) were reduced after TAD, while increased after TWD for both AA and CA. The transition of diet changed the levels of leptin (AA: P = 0.012; CA: P = 0.002) and PAI-1(P = 0.033) significantly for AA and CA. Significant changes for adiponectin (P = 0.033) and isoprostane (P = 0.009) were only observed in CA when they transitioned from TAD to TWD.

**Table 3:** Physiological Responses to Different Diets among Asian Americans and Caucasian Americans in Intervention Group, including drop-outs (Intent-to-treat).

	Asian Americans (N = 23)					Caucasian Americans (N = 18)				
	TAD^a^	P-value^b^	TWD	P-value^c^	P-value^d^	TAD	P-value^b^	TWD	P-value^c^	P-value^d^
	ΔVisit 3-Visit 2		ΔVisit 4-Visit 3			ΔVisit 3-Visit 2		ΔVisit 4-Visit 3		
**Weight (kg)**	−1.8±1.6	<0.001	0.3±0.9	0.131	<0.001	−1.4±1.4	0.003	0.9±1.5	0.028	0.001
**BMI (kg/m^2^)**	−0.6±0.6	<0.001	0.1±0.3	0.283	<0.001	−0.5±0.5	0.001	0.3±0.6	0.058	0.002
**Body Fat %**	−1.5±1.6	<0.001	0.7±1.2	0.016	<0.001	−1.9±1.5	0.001	1.2±1.7	0.017	0.001
**Trunk Fat %**	−2.1±2.0	<0.001	0.9±1.8	0.019	0.001	−2.4±1.8	0.001	1.4±2.1	0.016	0.001
**Waist-to-hip ratio**	0.0±0.0	0.295	0.0±0.0	0.795	0.562	0.0±0.0	0.056	0.0±0.0	0.073	0.052
**BP systolic (mmHg)**	1.1±15.2	0.831	−3.2±8.2	0.112	0.472	2.3±10.1	0.527	−3.3±6.9	0.098	0.184
**BP diastolic (mmHg)**	0.3±10.5	0.936	−1.1±9.7	0.602	0.667	−0.9±6.8	0.528	1.9±5.1	0.235	0.344
**Insulin AUC (µU/mL × h)**	−1402.4±2320.8	0.017	606.2±1898.9	0.232	0.015	−466.0±587.1	0.008	223.5±1094.4	0.650	0.034
**Glucose AUC (mg/dL xh)**	−1361.1±2397.9	0.025	−109.6±2476.5	0.896	0.149	−626.5±1691.4	0.255	−228.3±1537.5	0.463	0.433
**HOMA-IR**	−0.3±0.8	0.099	0.2±0.7	0.232	0.042	−0.1±0.3	0.101	0.0±0.3	0.753	0.221
**Total Cholesterol (mg/dL)**	−23.9±23.7	0.001	17.9±20.5	0.001	0.001	−27.6±24.8	0.001	23.3±22.1	0.002	0.001
**Triglycerides (mg/dL)**	15.2±62.7	0.526	27.9±126.3	0.778	0.765	2.4±20.5	0.600	−5.3±17.3	0.249	0.289
**HDL (mg/dL)**	−9.5±9.4	0.001	6.2±7.1	0.002	<0.001	−11.6±9.9	0.001	10.8±10.2	0.002	0.001
**LDL (mg/dL)**	−14.0±21.3	0.005	5.8±14.8	0.054	0.014	−14.9±15.6	0.003	11.1±14.4	0.009	0.001
**Microalbumin** **(µg albumin/mg creatinine)**	−1.7±7.9	0.313	−1.4±11.6	0.332	0.170	−2.1±7.7	0.136	1.7±8.2	0.583	0.239
**%B**	0.8±2.9	0.192	−0.8±2.5	0.117	0.154	1.4±2.9	0.046	−0.2±1.9	0.650	0.196
**%N**	−1.5±4.1	0.079	0.7±3.7	0.351	0.131	−1.5±2.9	0.055	1.4±3.6	0.117	0.041
**CRP (ng/mL)**	−50.7±1075.9	0.639	−74.8±1080.6	0.627	0.741	−566.8±3102.5	0.345	−348.1±2295.3	0.552	0.552
**Leptin (ng/mL)**	−2.2±3.7	0.014	1.6±3.3	0.016	0.012	−3.3±4.3	0.006	2.8±6.1	0.023	0.002
**Adiponectin (ng/mL)**	−1071.8±5947.3	0.520	1344.7±7465.1	0.370	0.205	−2612.4±5224.1	0.046	4836.4±9269.1	0.033	0.033
**PAI-1 (ng/mL)**	−13.4±30.4	0.012	−8.7±53.0	0.218	0.027	−2.0±4.9	0.173	3.8±8.9	0.028	0.033
**IL-1B (serum)(pg/mL)**	2.4±7.6	0.205	6.1±6.8	0.002	0.192	2.7±4.2	0.041	2.2±4.6	0.050	0.814
**IL-6 (serum)(pg/mL)**	0.3±2.2	0.394	0.0±1.8	1.000	0.639	0.0±1.4	1.000	−0.2±1.9	0.695	0.875
**TNF-α(pg/mL)**	−0.2±5.8	0.689	0.1±4.3	0.664	0.357	0.6±5.1	0.433	−0.1±4.4	0.754	0.530
**IL-8 (pg/mL)**	−0.3±7.0	0.556	−2.9±4.8	0.030	0.455	3.1±6.6	0.158	0.5±6.4	1.000	0.433
**Isoprostane (ng/mL)**	0.1±1.8	0.664	0.9±1.7	0.014	0.590	−0.6±1.4	0.196	0.8±1.8	0.055	0.009

a BP, blood pressure; CRP, C-reactive protein; HOMA-IR, Homeostatic model assessment-insulin resistance; OGTT, oral glucose tolerance test; PAI-1, plasminogen activator inhibitor-1; % B, endothelium-mediated dilation; % N, non-endothelium-mediated dilation; IL, interleukin; TAD, traditional Asian diet; TNF-α, tumor necrosis factor-alpha; TWD, typical Western diet.

b P-value of the changes observed before and after 8 weeks of TAD (Visit 3-Visit 2).

c P-value of the changes observed before and after 8 weeks of TWD (Visit 4-Visit 3).

d P-value of the Visit 4-Visit 3 and Visit 3-Visit 2.

Analysis of subjects who did not experience significant weight loss when on TAD (N = 5) also exhibited a significant decrease in insulin AUC −1259.5±2082.0 µU/mL×h (P = 0.043).

Weight loss during TAD had a moderate but significant correlation with changes in body fat (R^2^ = 0.415, P<0.001), even after accounting for age and gender (R^2^ = 0.403, P<0.001). However, weight loss was not significantly correlated with glucose AUC, insulin AUC or HOMA-IR.

Of the 32 general health survey that were collected, 12 (37.5%) reported an improvement in health after consuming TAD, 19 (59.4%) felt their health was about the same, and one (3.1%) felt their health was not as good as before consuming TAD (P<0.001). After consuming TWD, of the 31 received surveys, only one (3.2%) reported an improvement in their health, 23 (74.2%) reported their health was about the same, and seven (22.6%) felt their health worsened compared to the beginning of TWD (P<0.001).

Comparing the self-reported energetic level changes after the TAD and TWD, of the 32 responses, six (18.8%) felt more energetic after transitioning to TAD, 16 (50.0%) felt their energy level was about the same, and ten (31.3%) felt less energetic (P<0.001). After transitioning to TWD, eight of the 31 respondents (25.8%) felt more energetic, 16 (51.6%) felt about the same, and seven (22.6%) felt they had less energy (P<0.001).

In the 32 palatability surveys received, 19 (59.4%) reported that TAD tasted good or excellent, while 12 (37.5%) thought that TWD tasted good or excellent. Twenty-three (71.9%) and 20 (62.5%) felt that TAD and TWD were easy or very easy to follow, respectively. For both TAD and TWD, 28 (87.5%) felt satisfied after eating the meals.

## Discussion

In this randomized control pilot feasibility study using isoenergy TAD and TWD, the results showed that the macronutrient composition of TAD improved insulin sensitivity in both AA and CA. In contrast, TWD may have impaired the insulin sensitivity in AA, but not CA with normal BMI, raising the possibility that diets may elicit different effects in different ethnic groups.

Despite high dietary adherence, steady activity level, and the strenuous effort to adjust energy every two weeks to maintain an isoenergy state, a modest weight loss (2–3%) was observed in both AA and CA during TAD, which was not observed during TWD. This is consistent with the decrease in BMI, body fat and trunk fat for both AA and CA from TAD. One potential explanation for the weight loss could be the result of participating in a study, a known phenomenon in subjects modifying their behavior while being studied [24]. Another possible explanation could be the increase in fiber, which can contribute to the feeling of satiety and lead to decreased consumption [25]. Interestingly, for the participants in the control group who stayed on the TAD throughout the entire study, an additional 119.8 kcal/day was provided during the second 8 weeks of TAD to maintain weight. With an extra 6,708 kcal over the last 8 weeks of the study, 0.9 kg weight increase would be expected, instead, weight loss of −0.4 kg was observed. This observation raises the possibility that consumption of TAD may facilitate weight loss, which could be due to the high fiber content of the diet [25]–[27]. Further study of TAD on a weight loss goal is needed to clarify the factors that are enabling weight loss while on this diet.

One of the most striking effects of TAD was the significant improvement in insulin sensitivity (decreased insulin AUC), and improvement in glucose metabolism (decreased glucose AUC) observed among AA with high risk for diabetes. These improvements were not correlated to the modest weight loss resulting from TAD, as determined by the results in the regression analysis. Most studies have shown that an improvement in insulin sensitivity is only observed after 5% weight reduction [28], [29]. In our present study, the improvement was seen with only 3% weight loss. In addition, insulin AUC significantly decreased, even among participants who did not lose weight, suggesting factors other than weight loss may improve insulin action due to TAD. The combination of high fiber and low fat composition in TAD may also improve insulin sensitivity, since a high fiber diet has been reported to decrease glucose absorption, improve insulin response and glycemic control [30], [31]. The lower fat content of TAD than TWD further improves insulin sensitivity by lowering free fatty acids, which are known to induce insulin resistance by inhibiting insulin metabolic signaling pathways [32].

TAD also improved the lipid profile of all participants. Total cholesterol and LDL levels were significantly decreased, which is likely due to the high fiber and low fat composition of TAD [33], [34]. Although HDL level was also lowered, the HDL:LDL ratio was not changed significantly. The change in HDL is likely due to a reduction in fat consumption, including monounsaturated and polyunsaturated fats that have been known to raise HDL. This observation is consistent with findings from other low fat, high carbohydrate diet studies [31], [35].

Consumption of TAD significantly decreased indications of systemic inflammatory markers such as leptin and PAI-1 for AA. Although limited by the small sample size, most inflammatory markers showed an improving trend. Since increases in inflammation can induce insulin resistance and is associated with increased risk of type 2 diabetes [36], the reduction in inflammatory cytokines while on TAD suggests its potential in lowering the risk for type 2 diabetes. Future studies with more participants will be beneficial in confirming these findings.

In contrast to TAD, subjects on TWD had a significant weight gain and increase of body fat, especially trunk fat. Among AA, despite an insignificant weight gain, there was an elevation in body fat and trunk fat, which were consistent with a rise in insulin AUC, and HOMA-IR. These changes suggest that TWD increased levels of insulin resistance and the risks for diabetes and cardiovascular disease for AA even if it did not lead to significant weight gain. Surprisingly, CA had a significant increase in weight, body fat and trunk fat, yet no significant change in insulin resistance was observed. This difference between CA and AA could be due to increased insulin resistance in AA from small elevations of BMI, even at BMI of around 23. In contrast to other ethnic groups, increased risk of type 2 diabetes and loss of β-cell function are observed with BMI >27 [37].

In addition, consumption of TWD worsened lipid profiles and elevated inflammatory markers for both AA and CA. Statistically significant increases in total cholesterol, LDL, IL-1B, IL-8 and isoprostane were observed in AA and elevation of PAI-1 and IL-1B for CA. The small sample size in our study does not allow us to generalize our findings; however, these changes are consistent with findings from other studies [38], which also reported elevation of inflammatory markers and risk of cardiovascular disease with TWD.

Epidemiologically, the introduction of TWD, in the form of fast food, into Asia has been suggested to contribute to the surge in obesity and diabetes [39]. Our study provides evidence that the increase risk of diabetes from TAD-TWD transition may be partly independent of weight gain. It also suggests that AA and CA may respond differently when they are exposed to TWD. Increase in insulin resistance was noted in AA after TWD, but CA with normal BMI appeared to be protected when they transitioned from TAD to TWD. Future larger scale studies will be needed to confirm the findings in the different responses between AA and CA to the change of the diet.

Recent studies have suggested that high carbohydrate diets have contributed to the obesity epidemic and worsening of glucose metabolism [40], [41]. Our study shows that a high carbohydrate meal consisting of high fiber and low fat content reduces the risks of diabetes and cardiovascular disease in both AA and CA, and helps reduce weight and body fat.

TAD has been considered as a diet consumed by people in the lower socioeconomic strata and has not been embraced by the public in Asia. When modern ingredients and cooking techniques were used to prepare our study meals with the macronutrient composition of TAD, participants found that the meals were palatable and perceived them as beneficial for health. Compared to the published adherence rates of four other popular diets (Atkins, Zone, Weight Watchers and Ornish), our diet has a comparable, if not better adherence [42]. The advantages of TAD are its greater flexibility to choose a variety of foods, since no food group is drastically limited, and its lower cost compared to a high protein diet.

Limitations to this study include a small sample size, short intervention period and possible recall error in reporting the food consumption. With the small sample size, we were not able to establish longitudinal modeling to observe the changes in biomarkers in a time trend. The drop-out rate of this study was high, but it was comparable to other nutrition intervention studies[42]–[44]. Although our sample was not representative of the entire population, it is alarming that young AA demonstrated an adverse metabolic profile even at BMI <23 kg/m^2^ at the baseline, and after the TAD-TWD transition. Our study was limited to AA from East Asia since genetically they are similar but as a group different from South Asian Americans. Although the clinical phenotype of type 2 diabetes such as low BMI are very similar between East and South Asian Americans, the results reported here need to be confirmed in South Asian Americans. Diabetes is one of the fastest growing non-communicable diseases in the world. In this study, AA with normal BMI were shown to have elevated risks for developing diabetes to a much greater degree than CA. In contrast to previous reports, we have shown that the changes in diet patterns may affect AA and CA populations differently. Although Asians are prone to be at greater risk of diabetes than CA, the disease is preventable as shown in multiple large studies [45]. In summary, our study has shown that TAD with its macronutrient composition promotes weight loss, reduces insulin resistance, and could be considered as a strategy to prevent the epidemic of obesity and type 2 diabetes in Asian and Caucasian populations at risk for diabetes.

## Supporting Information

Checklist S1 **CONSORT Checklist.**
(DOC)

Protocol S1 **Trial Protocol.**
(DOC)
