# Supplementary material for: Improvement of Insulin Sensitivity by Isoenergy High Carbohydrate Traditional Asian Diet: A Randomized Controlled Pilot Feasibility Study
Source: PLoS One. 2014 Sep 16;9(9):e106851. doi: 10.1371/journal.pone.0106851 (PMC4167335; doi:10.1371/journal.pone.0106851)
Supplement: Protocol S1 — Trial Protocol. (DOC) [file pone.0106851.s002.doc]

# JOSLIN DIABETES CENTER

*Committee on Human Studies*

Application for Review and Approval of Research

and Training Projects Involving Human Research

**Principal Investigator:** George L. King, MD

**Co-Investigator(s):** William C. Hsu, MD; Sophia Cheung, MS, RD; Peggy Leong, DMD; Hillary Keenan, PhD; Allison B. Goldfine, MD; David Kim, DMD, Justine Hsu, Nancy Hong

**Project Title:** A pilot study of the changes in inflammatory state in Asian Americans transitioning from traditional Asian diet to typical American diet

**Funding:** Internal, Asian American Diabetes Initiative

**Study Contact:** Nancy Hong, [*Nancy.Hong@joslin.harvard.edu*](mailto:Nancy.Hong@joslin.harvard.edu) , 617-732-2606

**PURPOSE OF PROTOCOL:**

1. To compare the inflammatory responses (primarily PKC activation in monocytes), between Far-East Asian Americans and Caucasian Americans, when they change from a traditional Asian diet to a typical American diet.
2. To correlate the biochemical changes of inflammatory responses in the plasma and monocytes with those in the gingival crevicular fluid (GCF).

**STUDY DESIGN:**

This is an 18-week (2 weeks run-in and 16 weeks diet intervention), randomized controlled study involving 50 non-diabetic subjects with family history of diabetes. Subjects can be normal glycemic, have family history of impaired fasting glucose, gestational diabetes or have impaired glucose tolerance but cannot have frank diabetes, defined by the American Diabetes Association Clinical Guidelines*[[1]](#endnote-2)*. Family history of diabetes is defined as subjects having a first degree relative and grandparents diagnosed with diabetes mellitus. One half of the subjects (n = 35) will be Asian Americans of Far-East Asian decent (Korean, Japanese, Chinese) while the other half (n = 35) will be Caucasian-Americans. All subjects will follow 8 weeks of Asian diets (Visit 2) after 2 weeks of run-in period. At Visit 3, subjects will be randomized. 10 out of the 35 subjects in the Asian group and 10 out of the 35 subjects in the Caucasian group will be randomized to continue following Asian Diet (AD) for another 8 weeks, serving as controls. The remaining 25 individuals, in both the Asian and Caucasian cohorts, will be randomized to switching to 8 weeks of Western Diet (WD). Eight weeks of controlled diet has been shown in previous studies to be adequate in detecting differences in blood lipids and blood pressure.*[[2]](#endnote-3)*

| **Asian (n = 25)** | | **Caucasian (n = 25)** | |
| --- | --- | --- | --- |
| **Intervention Diet**  **(N = 20)** | **Control Diet**  **(N = 5)** | **Intervention Diet**  **(N = 20)** | **Control Diet**  **(N = 5)** |
| AD for 8 weeks | AD  (16 weeks) | AD 8 weeks | AD  (16 weeks) |
| WD for 8 weeks |  | WD 8 weeks |  |

**AD** = Asian Diet; **WD** = American (Western) Diet

**Table 1** is a graphical presentation of study design.

The dietary composition of this study was designed to reflect the proportion of the various macronutrients in traditional Asian and Westernized diets although the actual foods or combination of foods are not necessarily the same as those found in traditional rural Asian (AD) or typical Western diets (WD).

**Asian Diet (AD)**

- About 70% carbs, 15% protein, 15% fat (20% sat fat) less than 1% trans fats, 15g

fiber/1000kcal

- Caloric distribution: 25% breakfast, 40% lunch, 25% dinner, 10% after dinner/bedtime snacks
- For details, please see study summary flow sheet

**Western/ American Diet (WD)**

- About 50% carbs, 16% protein, 34% fat (20% sat fat), 4% trans fats, 6g fiber/1000kcal
- Caloric distribution: 25% breakfast, 40% lunch, 25% dinner, 10% after dinner/bedtime snacks
- For details, please see study summary flow sheet

Veronique Corporation will prepare and package all the diets for the study. Raw ingredients and commercially prepared food products will be provided by Veronique Corporation and Sunstar Inc. in Japan. The research dietician will work with a chef with a specialty in Japanese, Korean and Chinese cuisines to increase palatability of meals served. To ensure quality of the meals, Tokyo City, Yang’s Inc. will prepare and deliver a test meal once a month for food tasting by research staff members. Study investigators will also visit the facility to inspect the operations and document the findings on a monthly basis.

Subjects will be instructed to consume only the 3 meals and one snack a day provided except for non-sugar containing beverages. A list of snacks allowed will be provided. Subjects’ dietary habits will also be assessed by 24-hr dietary recall. If they consume alcoholic or caffeinated beverages, they will be advised to maintain their usual intake record consumption of these beverages during the study. Subjects will be provided instructions about compliance at the beginning of the study and reinforced throughout the study. They will be asked to maintain their weight and keep their physical activities constant throughout the study. Complete consumption of food provided is highly encouraged and any leftovers will be recorded. Subjects will estimate the percentage of each food item that is not consumed and record this information on a weigh-back form. A dietitian will briefly meet with each subject bi-weekly to measure weight, adjust caloric intake as necessary, ensure compliance, and answer any questions subjects may have. Caloric intake of +/- 250 kcals will be adjusted bi-weekly for subjects with weight change of more than +/- 2 kg from the baseline weight.

Subjects will be scheduled to 4 main visits: **Visit 1** - Screening, **Visit 2** – Asian Diet phase, **Visit 3** – AD or WD phase, and **Visit 4** – final visit. At these visits, anthropometric measurement, lab assessments and DEXA scan will be performed. All subjects will undergo a dental screening questionnaire at the beginning of the study to assess gum health. Subjects will also be scheduled to 6 additional visits to Joslin for weight measurement. All subjects will undergo periodontal evaluation and gingival crevicular fluid (GCF) extraction at each visit at Harvard School of Dental Medicine and Doppler forearm Sonography will be performed at Joslin Diabetes Center.

##### Details of Subjects

A thorough search of the patient database (IDX and NextGen) at the Joslin Clinic and the Asian Clinic at Joslin will be undertaken to identify subjects with history of diabetes who have not, according to Joslin records, voluntarily opted out to be contacted for potential research studies. Since we are not studying diabetic individuals but individuals with family history of diabetes and medical history of IGT, IFG and GDM, we will contact these individuals to ask their family members who may be interested to contact us for screening. In addition, we will enlist eligible Far East Asian Americans from: local Asian community health centers, from various college campuses and Asian American organizations. This will be done through advertisements placed on bulletin boards at medical centers, schools, or through referrals from colleagues. Subjects will also be recruited from fliers, advertisements, or through posting on the Internet. Invitation letters will be sent to eligible subjects. Advertisement samples will be presented for IRB approval prior to use. Subjects will receive informed consent form via mail prior to the initial visit. During the initial contact, a study investigator will explain the purpose, nature, and potential risks of the study in detail to the potential volunteer in languages they can understand. We plan to only include subjects who can understand English. If other Asian languages are needed to facilitate communication with subjects, they will be developed by professional translators and submitted for IRB approval. Brief questions will be asked to confirm demographic and medical information for eligibility. They will be given time to ask questions and make appointment for screening.

**Details of the Intervention Group(s)**

As discussed, subjects from both Asian American group and Caucasian American group will be randomized to **(a) Control Group (AD for 16 weeks)** or **(b) Intervention Group (AD for 8 weeks followed by 8 weeks of WD)**. At Visit 3, an envelope with the randomization assignment will be opened to help subjects determine the group they are assigned to. The **Control Group (b)** subjects will be fed AD for the entire 16 weeks. Subjects in the **(a) Intervention group** will be fed 8 weeks of AD followed by another 8 weeks of WD.

We plan to keep subjects’ physical activities and weight stable throughout the study since exercise and weight loss are reported to reduce inflammatory response in humans. Diets will be designed to maintain weight for the duration of the study. To ensure weight stability, subjects will be weighed bi-weekly and followed up with weekly phone call by a registered dietitian and are encouraged to discuss any dietary issues or concerns at that time. Caloric intake of +/- 250 kcals will be adjusted weekly for subjects with weight change of more than +/- 2 kg bi-weekly from the baseline weight. Subjects will be scheduled to 4 main visits: **Visit 1** - Screening, **Visit 2** - AD, **Visit 3** - WD or AD, and **Visit 4** - final. At these visits, we will perform anthropometric measurement, lab assessments, DEXA scan, Doppler forearm Sonography and dental evaluation. In addition, subjects are required to make 6 visits for weight measurement at Joslin. Vitamins and minerals intake will be assessed. Those who have not begun a multivitamin will be provided a multivitamin once daily.

**Details of Study Procedure(s)**

**Visit 1 – Screening (Week 0 – Week 2)**

Prior to Visit #1, all subjects will be screened by a checklist (base on inclusion/exclusion criteria listed in Part C). Each will be asked to fast before coming to the visit.

Eligible subjects will be scheduled for **Visit 1 (beginning of week 0)** with a study physician who will collect the consent forms from Joslin Diabetes Center and Beth Israel Deaconess Medical Center. Medical history, physical examination, anthropometric measurements, blood tests, blood pressure, pregnancy tests for females, and an oral glucose tolerance test will be performed after this. Those who have blood glucose results > 200mg/dl from oral glucose tolerance test will be excluded from the study. A registered dietician will perform a 24-hour diet recall, counsel the dietary procedures and use of accelerometer. Total Energy Expenditure (TEE) for each subject will be calculated to determine caloric intake using the following equation:

**Mifflin-St.Jeor equation:*[[3]](#endnote-4)***

**Men:** (10 x w) + (6.25 x h) - (5 x a) + 5

**Women:** (10 x w) + (6.25 x h) - (5 x a) - 161

W = weight (kg) h = height (cm) a = age

**Activity Factors:**

An activity factor of 1.35 will be used with Mifflin-St.Jeor equation to determine subjects’ initial total energy expenditure. This activity factor has been documented to be applicable for weight maintenance in healthy non-overweight adults.*[[4]](#endnote-5)* We will ensure weight stability by adjusting +/- 250 kcals each week for a weight change of +/- 2 kg from baseline weight per week.

Since subjects are encouraged to maintain weight, a self-administered questionnaire will be used to monitor their activity levels throughout the study.*[[5]](#endnote-6)* In addition, we will also use the Actiped to monitor physical activity levels of all subjects. This questionnaire will be given at Visit 2, 3 and 4.

All subjects will receive a dental examination and a full mouth dental x-ray at the HSDM. HSDM investigators will explain dental procedures in detail. Subjects will then be asked to sign the HSDM ICF. They will also complete questionnaire about general health and quality of life.

During these 2 weeks of screening period, subjects are required to maintain their usual weight and physical activities.

**Visit 2 - AD (Week 2 – Week 10)**

Subjects will be required to fast before this visit. Urine for albumin, blood tests, weight, anthropometric measurements, vital signs and DEXA scan will be performed. Subjects will be assessed for arterial endothelial function by forearm Doppler Sonography at Joslin Diabetes Center and baseline dental evaluation for periodontal diseases will be performed at the Harvard School of Dental Medicine (HSDM). (For specific dental procedures, please see “Dental Procedures” below).

The study dietician will provide instructions to all subjects on beginning 8 weeks of **Asian Diet (AD)** prepared by Veronique Corporation. Subjects are required to only consume food (3 meals and a snack/day) provided for the rest of the study (except for non-sugar, non-alcohol containing beverages, a list will be provided) and continue to keep food weigh-back sheets. Foods throughout the entire study will be prepared and packaged by Veronique Corporation. Complete consumption of food provided is highly encouraged and any leftovers will be recorded. Subjects will estimate the percentage of each food item that is not consumed and record this information on a weigh-back form. A dietitian will briefly meet with each subject bi-weekly to measure weight, adjust caloric intake as necessary, ensure compliance, and answer any questions subjects may have. Caloric intake of +/- 250 kcals will be adjusted bi-weekly for subjects with weight change of more than +/- 2 kg from the baseline weight.

**Visit 3 - WD or AD (Week 11 – 18)**

At Visit 3 (End of Week 10), all subjects will receive an envelope that will determine if subjects will be randomized to the control (continue another 8 weeks of Asian Diets) or the intervention group (switching to 8 weeks of Western Diets). Anthropometric measurement, DEXA scan, weight, OGTT, vital signs, and blood tests including inflammatory markers will be repeated. Requirements in this phase will be the same as Visit 2. Subjects will be asked to complete a questionnaire about General health and quality of life. All subjects will also complete a Diet Palatability Survey, receive a forearm ultrasound evaluation at Joslin Diabetes Center and dental examination at HSDM. All subjects will fast before coming to this visit. While the **Control group(a)** will continue on AD, the **Intervention group(b)** will switch from AD to 8 weeks of WD prepared and packed by Veronique Corporation. starting from this visit.

**Visit 4 Final (End of week 18)**

Anthropometric measurement, DEXA scan, OGTT, weight, vital signs, urine for albumin, and blood tests including inflammatory markers will be repeated. Subjects will be asked to complete questionnaires for general health and diet palatability. Subjects will also fast before this visit. Subjects will receive the last ultrasound evaluation at Joslin Diabetes Center and return to HSDM for their fourth dental visit where the final oral examination will be performed and documented.

All subjects, both group (a) and (b), will receive a free dental cleaning at Harvard School of Dental Medicine at Visit 4.

**Detailed Dental Procedures:**

**(**All dental evaluation/ procedures will be performed at Harvard School of Dental Medicine**)**

**Periodontal Examination**

At the **Screening (Visit 1)**, the two HSDM investigators will perform complete periodontal examinations and identifying appropriate sites for GCF to be drawn on all the participants that will include:

1. Hard tissue charting
2. Soft tissue evaluation
3. Probing Depth of pockets utilizing the Florida Probe to standardized periodontal pocket measurements
4. Attachment Loss measurements (CEJ to the depth of the pockets)
5. Mobility measurements
6. Gingival crevicular fluid (GCF) acquisition at 4 sites (mesial-buccal pockets in all first molars) for each patient.

(The GCF will be sent to Boston University Dental School to determine levels of cytokines, IL-1, IL-6, IL-8, and TNF-α)

**Full Mouth Dental X-ray Films (Visit 1 ONLY)**

All participants will be seen at the Radiology Department at HSDM for their first visit to obtain a set of full mouth dental x-ray films (16-18 small dental films)

**Periodontal Diagnosis**

The two HSDM investigators will review the physical oral examination data and the full mouth dental x-ray films to arrive at a periodontal diagnosis for each of the fifty participants.

Based on the American Dental Association Classification, the periodontal diagnosis of the fifty participants will be classified into:

Case Type I: Gingivitis

Case Type II: Early Chronic Periodontitis

Case Type III: Moderate Chronic or Aggressive Periodontitis

Case Type IV: Advanced Chronic or Aggressive Periodontitis

Case Type V: Refractory Chronic or Aggressive Periodontitis

Each participant will be informed of their periodontal diagnosis after their first HSDM visit (Baseline data).

After the completion of Visit 1, the participants return to HSDM for their second visit. At the second visit the physical oral examination (Steps 1-6) will be repeated and documented. Data from the second visit will be compared to baseline to ensure no major deterioration in periodontal condition has occurred. If acute periodontal infections occur while the patients are participating in this study, they will be treated at HSDM by the investigators at no charge to the patients.

After the completion of Visit 3, the participants will return to HSDM for the fourth dental visit where the final oral examination, involving Steps 1-6, will be performed and documented.

**Dental Prophylaxis**

At the conclusion of the study, HSDM’s Faculty Group Practice Dental Hygienists will offer all participants a free dental prophylaxis. If they choose this benefit, they will be scheduled for a standard visit with a HSDM dental hygienist.

**Details of Measurements:**

1. **Anthropometric measurements**- measurements of weight, height, waist, hip ratio
2. **Dual Energy X-Ray Absorptiometry (DEXA)**- to evaluate total and regional body fat mass. This measurement will follow standard procedure for body composition measurement and will be performed on Hologic Delphi-A 70372. (Only at End of week 2 (V2), End of 11 (V3), and End of week 18(V4))
3. **Fasting Lipid Panels**- Total cholesterol, HDL, LDL, TG
4. **Hypercoaguability** markers- plasminogen activator inhibitor – 1(PAI-1)
5. **Systolic/Diastolic Blood Pressure**
6. **Insulin Sensitivity** – HOMA-IR, Fasting insulin and glucose level, hemoglobin A1c
7. **Inflammatory markers**- PKC levels in monocytes, CRP, Isoprostane, RNA, IL-6, IL-8, TNF-α, and IL1-β.
8. Other **hormones** related to weight and insulin resistance: TSH (Sceening visit only), leptin, adiponectin, ghrelin.
9. **Dental** assessments and collection of GCF to be performed by Harvard School of Dental Medicine.
10. **Vascular functions**: forearm blood flow study by Doppler Ultrasound. Measurement of brachial artery diameter: Flow-mediated endothelium-dependent vasodilatation and endothelium-independent vasodilation will be assessed in the brachial artery using high resolution B-mode ultrasonography. An ultrasound scanner (Toshiba model SSH-140A) equipped with a high-resolution linear array transducer (7.5 mHz) will be used to image the brachial artery and thus enable measurement of its diameter and cross-sectional area. A longitudinal image (parallel to the artery) will be acquired just proximal to the antecubital fossa. The transducer will be positioned at 90 degrees to the vessel to acquire an image through the center line of the vessels so that the near and far wall interfaces will be clearly discernible. A simultaneous electrocardiographic signal will be recorded. The video output and electrocardiographic signal of the ultrasound machine will be connected to a computer equipped with a Data Translation frame grabber video card. The R wave on the electrocardiogram will be used as a trigger to acquire (digitize) frames. Digitized images at baseline and after intervention (e.g., reactive hyperemia, nitroglycerin) will be stored on the hard drive and backed up on removable media. Acquisition and analysis of the stored images will be performed using software designed for this purpose by Information Integrity, Inc. The vessel wall lumen interface is determined by derivative based edge detection following identification of the region of the anterior and posterior walls by the investigator. The maximum diameter of the vessel is then determined. This technique yields an interobserver variability of 0.05±0.16% and intraobserver variability of 0±0.15%.

This study will be performed at the Joslin Diabetes Center. The dental evaluation will be performed with investigators from Harvard School of Dental Medicine.

###### Study Procedures Flow Sheet

| **Study Visits** | **Procedures Checklist** |
| --- | --- |
| **Visit 1**  **Screening** (Wk 0-2)    Beginning of Week 0  About 5 hours | - *Complete Initial screening form and Dental screening form   (before the Visit 1)   - Fasting before Visit   (No food, no drink, except water for 10 hours before visit)   - Meet with **Physician** for   **- Collect the Joslin Diabetes Center Consent Form**  - Initial screening  - Explanation of the study   - Meet with **Nurse** for   - Vital Signs  - OGTT – test for diabetes  - Pregnancy test for women by obtaining a urine sample  - Blood draw   - Meet with **Dietician** for   - Measurements of weight, height, hip, and waist  - Nutrition assessment, discussion of intervention diets  - Discuss physical activity (PA),  - Ascertain history of food allergies   - Meet with **Investigator at HSDM** for   - Explaining dental procedures  **- Obtaining HSDM Consent Form**  - Full mouth dental x-ray   - * Complete General Health Questionnaire 1 - Free Parking as needed |
| **Visit 2**  **Asian Diets** for both Intervention **(b)** & Control **(a)**  (Wk 2-10)    End of Week 2  About 5 hours | - Fasting before visit   (No food, no drink, except water for 10 hours before visit)   - Meet with **Nurse** for   - Vital Signs  - Urine Protein test by obtaining a urine sample  - Blood draw – lipids, inflammatory markers   - Meet with **Dietician** for   - Measurements of weight, height, hip, and waist  - DEXA scan (to determine percentage of body fat)   - Meet with **Investigator at HSDM** for:   - Dental evaluation   - Compensation: $50.00 after completing this visit - Free Parking as needed - Forearm blood flow measurement (Doppler Sonography Ultrasound) - Randomization occurs at the end of week 10 |
| **Visit 3**  **WD** for intervention group  **AD** for control group  (Wk 10-18)  End of Week 10  About 5 hours | - Fasting before visit   (No food, no drink, except water for 10 hours before visit)   - Complete General Health Questionnaire 2 - Meet with **Dietician** for   - Measurements of weight, height, hip, and waist  - DEXA scan (to determine percentage of body fat)   - Meet with **Nurse** for   - Vital Signs  - OGTT – test for diabetes  - Urine Protein test by obtaining a urine sample  - Blood draw – lipids, inflammatory markers   - Meet with **Investigator at HSDM** for:   - Dental evaluation   - Compensation: $150.00 after completing this visit - Free Parking as needed - Forearm blood flow measurement (Doppler Sonography Ultrasound) - Complete Diet Palatability Survey |
| **Visit 4**  Final Visit  End of Week 18  Total Hours: About 5 hrs | - Fasting before visit   (No food, no drink, except water for 10 hours before visit)   - *Complete General Health Questionnaire 3 - *Complete Diet Palatability Survey - Meet with **Dietician** for   - Measurements of weight, height, hip, and waist  - DEXA scan (to determine percentage of body fat)   - Meet with **Nurse** for   - Vital Signs  - OGTT – test for diabetes  - Urine Protein test by obtaining a urine sample  - Blood draw – lipids, inflammatory markers   - Meet with **Investigator at HSDM** for:   - Dental evaluation  - Free dental cleaning for all subjects (both group **(a)** and **(b)**)   - Compensation: $300.00 after completing this visit - Free Parking as needed - Bilingual Guide – Staying Healthy with Diabetes - Forearm blood flow measurement (Doppler Sonography Ultrasound) |

###### *Please see appendix 3 for questionnaires and screening forms

###### INCLUSION / EXCLUSION CRITERIA

**Inclusion criteria:**

1. Caucasian or Far-East Asian decent
2. Ages between 25 – 55 years old
3. Has family history of diabetes defined as having a first degree relative diagnosed with diabetes mellitus and/or medical history of gestational diabetes (GDM), impaired fasting glucose (IFG) and impaired glucose tolerance (IGT)
4. BMI of 18.5 – 27.0 kg/m^2^
5. Agree to maintain constant physical activity levels for the duration of the study
6. Agree to utilize contraception for the duration of the study (for female subjects)
7. Have a minimum of 12 natural teeth
8. Scoring of 2 or more on dental screening questionnaire (See appendix)
9. Ability to communicate in English.

**Exclusion criteria:**

1. Acute weight loss/weight gain over the past 6 months (defined as +4 lbs or more/month)
2. History of diabetes
3. Current Smokers (including those who quit < 1 year)
4. Heavy alcohol drinkers, defined as drinking more than 7 drinks/week. Those who drink alcohol will need to be reported and documented. They will be asked to maintain the levels of alcohol consumption throughout the study.
5. Medical history of vascular diseases (CAD, MI, stroke, CABG, angioplasty), diabetes, liver disease, kidney disease, cancer, AIDS, bleeding disorders
6. History of food allergies or to any food products in the diet menu
7. Enrolled in another investigational study within 1 month prior to screening for this study
8. Vegetarian, those with significant food aversions, and anyone who cannot comply with the diet
9. Pregnancy or breasting feeding
10. Other autoimmune or inflammatory conditions or diseases that may compromise the patient's safety or compliance during the study on an individual basis
11. Chronic use of antibiotics or anti-inflammatory medications (>1 month) within the past year
12. On medications such as statins, angiotensin converting enzyme inhibitor, warfarin, aspirin or other anti-inflammatory medications. Daily multivitamins are permitted, excluding pharmacological doses of anti-oxidants will not be allowed.
13. *Individuals with dental needs requiring extensive dental restorations such as cavities, root canals, crowns, etc.*

## **Details of Outcome Measurements**

## OGTT, laboratory analysis of blood and screening for medical history will be performed to exclude patients with diabetes mellitus, food allergies and other chronic illnesses that will prevent them from participating safely in the study. OGTT, laboratory tests for inflammatory markers, lipids, glucose, thyroid functions, DEXA scan, forearm Doppler Sonography, and dental evaluation will be performed at baseline and measure outcomes of each diet phase. Questionnaires will also be used to assess subjects’ view of health status pre and post interventions. See study flow sheet above for types and frequency of measurements performed throughout the study.

###### DATA ANALYSIS / SUBJECT SELECTION

**Statistical Considerations**:

**Sample size and power calculations**:

This study is a pilot/ feasibility study. The primary hypothesis of this study is to compare changes in PKC activity due to the transition from a traditional Asian diet to a typical American diet in two different ethnic groups, Asian and Caucasian. The interest is the mean percent change in marker levels between the two ethnic groups, which would be the values at timepoint 3 (after exposure to American diet) minus the values at timepoint 2, divided by the measure at time point 2 times 100. This would give the percent change that could be accounted for by exposure to the different diets in each ethnic group. For this comparison, the power for comparing the net change between the two ethnic groups of 20 individuals who will change from a traditional Asian diet to an American diet was calculated. Additionally, two control groups (one of each ethnicity) will remain on the traditional Asian diet as a control for potential outside effects on the primary hypothesis, the power calculations for comparing the control group to the active group can also be found below.

A total of 25 individuals of each ethnic group will be randomized into either the traditional Asian diet group (n=5) for each period or into the traditional diet and then the American diet (n = 20). The total number of participants is limited by cost. It is our hope that the 5-person control arms makes it possible to assess whether a factor outside of diet contributed to any differences observed. These control groups of ten will only be used for a subset analysis to get an idea of possible environmental effects on the outcome of interest, change in PKC activity. An appropriate two-sample test will be used for this determination, with the alpha and power given in the proceeding paragraph.

The power for the examination of percent change in PKC activity, the primary hypothesis, was calculated using STATA 7.0 (College Station, Texas 2001). An assumption of a one-sided test with an alpha of 0.05 was made. Mean PKC measurements were estimated at 78.1*[[6]](#endnote-7)* ±5 (there is no expected ethnic variation in PKC activity levels). Calculations are based on change exhibited by cardiac cells with exposure to increased lipids. In previous trials a 50% change has been observed in PKC activity in the cytosol of cardiac cells.

Table 1. Power calculations for PKC activity percent change to detect a difference between Caucasian and Asian American groups when exposed to the American diet.

| **Size of Group 1** | **Size of Group 2** | **Percent change** | **PKC estimate** | **PKC Std dev (+)** | **Power** |
| --- | --- | --- | --- | --- | --- |
| 25 | 25 | 10 | 85.91 | 5.5 | 0.999 |
| 25 | 25 | 5 | 82.005 | 5.25 | 0.8527 |

This summary table of power to detect a significant difference suggests that a study using this approach would have adequate power to detect a difference between the two groups in PKC activity. We do not expect to see a difference less than 5%.

Table 2.  Power calculations for PKC activity change with change in diet based on a comparison to detect a difference between the control group and those exposed to the American diet. The baseline PKC activity value used in this calculation is 78.1 ±5.

| **Size of Group 1** | **Size of Group 2** | **Percent change** | **Comparison values** | **Power** |
| --- | --- | --- | --- | --- |
| 10 | 25 | 10 | 85.91 ±5.5 | 0.969 |
| 10 | 25 | 5 | 82.005 ±5.25 | 0.875 |

This demonstrates that we have adequate power to detect as low as a 5% change between the two groups. This exceeds our needs as estimated change is approximately 50%.

In addition to the primary endpoint of change in PKC levels, changes in Flow Mediated Dilation (FMD) and CRP are of interest. These differences will be examined by two-sample tests, either two-sample t or Wilcoxon sign rank. The power curves based on current literature for changes in FMD can be found below, based on a one-sided hypothesis using an alpha of 0.05, for the detectable difference of percent change between ethnic groups. The power to detect a difference in percent change for CRP measures was also examined. Our estimates indicate that there is insufficient power (~50%) to detect a difference as large as 25 between the two percent change measures. This was calculated assuming a one-sided test with an alpha of 0.05.

 Additionally, we present the power curve for the detection of difference in percent change in Flow Mediated Dilation.

Figure 1. Power curve for percent difference in FMD between Caucasians and Asians (the x-axis represents difference between the two measures) [[7]](#endnote-8),[[8]](#endnote-9) ,[[9]](#endnote-10),[[10]](#endnote-11)

From this graph it is evident that there will be sufficient power to detect a difference in FMD change between the two groups. [[11]](#endnote-12),[[12]](#endnote-13)

**Data Analysis:**

Our primary a priori hypothesis is to detect if there is a difference in PKC activity change between Caucasians and Asians with exposure to American diets, which will be done through a two-sample test (either t-test or Wilcoxon signed-rank sum, depending on distribution). A secondary hypothesis is to examine the difference in change of Flow Mediated Dilation before and after exposure to the American diets between the two ethnic groups, which will also be examined through a two-sample test. These will be examined with an alpha set at 0.05. Additionally, we expect to do several exploratory analyses, including anthropometrics, lipids, fibrinogen, IL-6, TNF-alpha, E-selectin, Isoprostane, Fox II, leptin, adiponectin, and ghrelin. We will use Scheffé’s procedure for linear contrasts, as the comparison of means is a special case of this to help correct for multiple comparisons. However, correction for multiple comparisons will be applied for all exploratory hypotheses.

Multiple potential confounding variables will be controlled for by including limitations on age, family history of diabetes, limited range of BMI (estimated body fat content), and physical activity. We will also take a diet history and exclude those with medical illnesses. These steps will not exclude all bias but should limit it in this study. The potential role of effect-modifiers will also be considered in the interpretation of the data. A preliminary stratified examination will be done to determine if there is a significant difference between exposed and unexposed groups of the same ethnicity. Baseline and post intervention measures permits change from baseline analysis. If change from baseline differs between groups, we are interested in designing future studies to identify the factors that mediate the differences in response between ethnic groups.

Additionally, an underpowered study of the correlation between GCF and inflammatory markers will be done using linear regression. As there are limited observations, only the idea of a trend is expected from this analysis. . It is recognized that several factors may differentially contribute to dental health, and could therefore, affect the findings of this analysis. An attempt to correct for these factors was made through the exclusion criteria, which rules out people who have smoked and those with other chronic diseases, which may be related to periodontal health. The age range of the population examined is also constrained to limit possible effect modification by age.

Data analysis will start with a univariate examination of variables, primarily for the determination of whether parametric or non-parametric statistics are appropriate. Hypothesis testing, for the primary hypothesis, will be undertaken using two sample t-tests or Wilcoxon Rank Sum test for continuous variables.

Due to the preliminary nature (pilot/ feasibility) of this study and small sample size, multivariable model building will only be done on an exploratory basis and is expected to be underpowered.

#### Local Interim Data Analysis/Data Monitoring Plan

**Data Safety Monitoring Plan**

Our data and safety-monitoring plan will involve monitoring by the principal investigator, a safety officer from the Joslin Diabetes Center, Robert Stanton, MD, along with our Institutional Review Board (IRB). The possible adverse events (AEs) that warrant reporting include significant symptomatic hypotension during the ultra-sound test following the administration of sublingual nitrates. In the protocol involving vascular ultrasound, sublingual nitroglycerine 0.4mg is administered to subjects who will assume the recumbent position while having his/her blood pressure monitored. The blood pressure will continue to be monitored when patient assumes the sitting position after 15 minutes of recumbence. The subject will return to recumbent position until the effect of nitroglycerine wears off if the subjects cannot tolerate the sitting or standing position. The DEXA scanning is considered non-invasive. All adverse events will be clearly documented in specific reporting forms, and reported by the principal investigator to the IRB and the Clinical Research Center (CRC). Adverse events will also be reported to the NIH Office of Biotechnology Activities (OBA) if required. In the event of any medical instability, the nurse coordinator will contact William Hsu, MD, 617-713-3473, or George King, MD 617-732-2622.

**POSSIBLE BENEFITS:**

There may not be any immediate benefit from participating in the study. However, the findings of the study may provide future directions on the prevention and lifestyle/medical treatment for diabetes in both Asian and Caucasian populations.

**POSSIBLE RISKS:**

Because this study primarily involves changes from one typical diet to another, we expect a very low risk to benefit ratio.

In this study the risk and discomfort may include:

**Oral Glucose Tolerance Test (OGTT)**

Subjects will be asked to drink a beverage with 75 g of glucose. Slight nausea may be experienced due to the concentration of sugar in the test medium.

**Blood Loss/Blood draw**

The total amount of blood drawn during the study is about 2 fluid ounces. This amount should not pose any risk to subjects.

**DEXA Scan**

The risk associated with the body composition test includes some exposure to low-level radiation. Exposure to radiation from a whole body DEXA for body composition is not excessive. While no amount of radiation has been proved to be safe, there is no direct evidence that the small doses of radiation used in this study, similar to those used in diagnostic radiology, cause harmful effects in the person who is exposed. The total effect of radiation dose from the DEXA test is less than 5 percent of the effective natural background radiation a person receives from living in a typical American community for one year. It is about the same radiation as that received from three coast-to-coast airline flights.

Lying still on a firm surface for a total of 20 minutes may cause minor discomfort.

**Ultrasound**

The procedures in the vascular study involve stopping the blood flow in the subject's forearm with a blood pressure cuff for 5 minutes. This may cause some numbness or discomfort in the arm. This will disappear soon after the cuff is released. Occasionally, minimal bruising occurs in the area where the cuff was inflated. This will disappear in a few days.

Subjects will be given Nitroglycerin during the blood vessel ultrasound exam, which can cause slight headache or dizziness. These reactions are usually temporary and do not require any formal treatment. For people taking Viagra, Cialis or Levitra the use of these medications may cause a harmful interaction with the nitroglycerin used in the ultrasound study, there we advise subjects not to take Viagra for 24 hours and Cialis and Levitra for 72 hours before participating in the ultrasound test. On the day of the test, we will ask the subject if he or she is taking Viagra.

**Asian Diets (AD) and Western/ American Diets (WD)**

Participants may expect a higher lipid profiles and blood glucose levels on either diet.

**Unforeseen Risks**

There may be other risks not yet identified

Adequacy of Protection Against Risks:

Procedures to protect against potential risk:

**OGTT**- The test medium will be pre-chilled to minimize nausea

In the protocol involving vascular ultrasound, sublingual nitroglycerine 0.4mg is administered to subjects who will assume the recumbent position while having his/her blood pressure monitored. The blood pressure will continue to be monitored when patient assumes the sitting position after 15 minutes of recumbence. The subject will return to recumbent position until the effect of nitroglycerine wears off if the subjects cannot tolerate the sitting or standing position. The possible adverse events (AEs) that warrant reporting include significant symptomatic hypotension during the ultra-sound test following the administration of sublingual nitrates.

The DEXA scanning is considered non-invasive. All adverse events will be clearly documented in specific reporting forms, and reported by the principal investigator to the IRB and the Clinical Research Center (CRC). Adverse events will also be reported to the NIH Office of Biotechnology Activities (OBA) if required. In the event of any medical instability, the nurse coordinator will contact William Hsu, MD, 617-713-3473, or George King, MD 617-732-2622.

**RECRUITMENT AND CONSENT PROCEDURES:**

A thorough search of the patient database (IDX and NextGen) at the Joslin Clinic and the Asian Clinic at Joslin will be undertaken to identify subjects with history of diabetes who have not, according to Joslin records, voluntarily opted out to be contacted for potential research studies. Since we are not studying diabetic individuals but healthy individuals with family history of diabetes, we will contact these individuals to ask their family members who may be interested to contact us for screening. In addition, we will enlist eligible Far East Asian Americans from: local Asian community health centers, from various college campuses and Asian American organizations. This will be done through advertisements placed on bulletin boards at medical centers, schools, or through referrals from colleagues. Subjects will also be recruited from fliers, advertisements, or through posting on the Internet. Invitation letters will be sent to eligible subjects. Advertisement samples will be presented for IRB approval prior to use. Subjects will receive informed consent form via mail prior to the initial visit. During the initial contact, a study investigator will explain the purpose, nature, and potential risks of the study in detail to the potential volunteer in languages they can understand. We plan to only include subjects who can understand English. If other Asian languages are needed to facilitate communication with subjects, they will be developed by professional translators and submitted for IRB approval. Brief questions will be asked to confirm demographic and medical information for eligibility. They will be given time to ask questions and make appointment for screening. Both genders are elicited equally to participate in the study.

***RIGHTS AND PRIVACY:***

**Please answer the following questions:**

- Will medical history/clinical information be obtained from the subjects’ medical records for the purpose of this study? If yes, please list what information will be recorded.

**X YES.**

List of information recorded:

- Anthropometrics
- Contact information
- Medical history
- Medications
- Lab results
- Will information resulting from this study (i.e. results of clinical/research lab tests, etc…) become part of the subjects’ medical record or provided to the subject and/or others for clinical purposes? If, no, please list what information will not be given to the subject or recorded in their medical record and why.

**X NO**, Ultrasound, Dexa, and specialized lab results will not be filed at subjects medical records because they may not have clinical relevance.

- Will subjects’ identifiable health information***** be shared with others outside of Joslin Diabetes Center? If yes, list whom this information will be shared with (please be specific, include names of collaborators, study sponsor contacts)?

**X YES**

Yes, the list will be shared with co-investigators from Harvard School of Dental Medicine since the dental intervention will be performed there.

***OMIT PROCEDURES / LEAVE STUDY:***

This is a voluntary study. Subjects have the right to omit specific procedures or to leave the study at any time during the study period. In cases of withdrawal or omitting certain procedures from the study, subjects are required to inform or/and provide a written and dated notice of the decision to the principal investigator or designated staff of the study.

***INCENTIVES / REMUNERATION:***

Subjects will receive a total $500 for participating in this study. They will first receive $50 after completion of visit 2; $ 150 after completion of visit 3 and $300 and a Bilingual Diabetes Guide (Chinese/English) after completion of Visit 4 (end of week 18). In addition, a total of 4 months of food will be provided to all subjects for the duration of the study. Free parking available upon each visit at Joslin (A total of 4 study visits and 6 short visits for weight measurement).

*** Identifiable Health Information**

Data that includes any of the following identifiers are considered identifiable health information:

- Name
- Social Security number
- Medical Record Number
- Address by Street Location
- Address by Town/City/Zip Code
- Date of Birth
- Admission or Discharge Date
- Date of Death
- Telephone Number
- Fax Number
- Electronic E-Mail Address
- Web URLs
- Internet Protocol (IP) Address
- Health Plan Beneficiary Number
- Account Number
- Certificate/License Number
- Vehicle Identification Number and Serial Number, including License Plate Number
- Medical Device Identifiers and Serial Numbers
- Biometric Identifiers (finger and voice prints)
- Full Face Photographic Image
- Any Other Identifier likely to identify the subject

***Please answer the following questions:***

1. Where and how will your project utilize the Joslin Diabetes Center?

**X CTU**

1. Will your project involve research on living human fetuses?

**No**

1. Does your project involve the use of any new drug or device?

**No**

1. Is review required by risk management foundation?

**No**

______________________________________________________ ____________________

***Signature of Principal Investigator Date***

**I have read and reviewed this application for approval by the Committee on Human Studies**

_______________________________________________________ ____________________

***Signature of PI’s Section Head Date***

Please bring the original and twenty-two (22) copies of

this form and the informed consent form for the above research project to

Leigh Read in the Office of Sponsored Research

by the appropriate CHS meeting deadline.

1. American Diabetes Association. **Diagnosis and Classification of Diabetes Mellitus.** Diabetes Care 2005 28: S37-42 [↑](#endnote-ref-2)
2. Obarzanek E, **Effects on blood lipids of a blood pressure-lowering diet: the Dietary Approaches to Stop Hypertension (DASH) Trial.** AJCN. 2001; 74:80-9. [↑](#endnote-ref-3)
3. Mifflin, et al. A new predictive equation for resting energy expenditure in healthy individuals. Am J clin Nutr 1990;51:241-7. [↑](#endnote-ref-4)
4. www.nap.edu/openbook/0309046335/html, copyright 1989, 2000. The National Academy of Sciences pg 26-30. [↑](#endnote-ref-5)
5. <http://www.ipaq.ki.se/dloads/IPAQ_SHORT_LAST_7_SELF_ADM-revised_8-23-02.pdf> [↑](#endnote-ref-6)
6. Ceolotto G, **Protein kinase C activity is acutely regulated by plasma glucose concentration in human monocytes in vivo.** Diabetes. 1999 Jun;48(6):1316-22. [↑](#endnote-ref-7)
7. [Williams MJ](http://www.ncbi.nlm.nih.gov.ezp1.harvard.edu/entrez/query.fcgi?holding=hulib&db=pubmed&cmd=Search&term="Williams+MJ"%5BAuthor%5D), [Sutherland WH](http://www.ncbi.nlm.nih.gov.ezp1.harvard.edu/entrez/query.fcgi?holding=hulib&db=pubmed&cmd=Search&term="Sutherland+WH"%5BAuthor%5D), [McCormick MP](http://www.ncbi.nlm.nih.gov.ezp1.harvard.edu/entrez/query.fcgi?holding=hulib&db=pubmed&cmd=Search&term="McCormick+MP"%5BAuthor%5D), [Yeoman DJ](http://www.ncbi.nlm.nih.gov.ezp1.harvard.edu/entrez/query.fcgi?holding=hulib&db=pubmed&cmd=Search&term="Yeoman+DJ"%5BAuthor%5D), [de Jong SA](http://www.ncbi.nlm.nih.gov.ezp1.harvard.edu/entrez/query.fcgi?holding=hulib&db=pubmed&cmd=Search&term="de+Jong+SA"%5BAuthor%5D). Phytother Res. 2005 Apr;19(4):314-9. **Aged garlic extract improves endothelial function in men with coronary artery disease**. [↑](#endnote-ref-8)
8. [Broadley AJ, Korszun A, Abdelaal E, Moskvina V, Jones CJ, Nash GB, Ray C, Deanfield J, Frenneaux MP.](http://www.ncbi.nlm.nih.gov.ezp1.harvard.edu/entrez/query.fcgi?holding=hulib&cmd=Retrieve&db=pubmed&dopt=Abstract&list_uids=16022966&query_hl=5) **Inhibition of cortisol production with metyrapone prevents mental stress-induced endothelial dysfunction and baroreflex impairment.** J Am Coll Cardiol. 2005 Jul 19;46(2):344-50. [↑](#endnote-ref-9)
9. [Beishuizen ED, Jukema JW, Tamsma JT, van de Ree MA, van der Vijver JC, Putter H, Maan AC, Meinders AE, Huisman MV.](http://www.ncbi.nlm.nih.gov.ezp1.harvard.edu/entrez/query.fcgi?holding=hulib&cmd=Retrieve&db=pubmed&dopt=Abstract&list_uids=15983319&query_hl=6) **No effect of statin therapy on silent myocardial ischemia in patients with type 2 diabetes without manifest cardiovascular disease**.Diabetes Care. 2005 Jul;28(7):1675-9. [↑](#endnote-ref-10)
10. Vasc Med. 1997;2(2):87-92. **Noninvasive assessment of endothelium-dependent flow-mediated dilation of the brachial artery.** Uehata A, Lieberman EH, Gerhard MD, Anderson TJ, Ganz P, Polak JF, Creager MA, Yeung AC. Cardiovascular Division, Brigham and Women's Hospital, Harvard Medical School, Boston, Massachusetts, USA. [↑](#endnote-ref-11)
11. [Santos AC, Lopes C, Guimaraes JT, Barros H.](http://www.ncbi.nlm.nih.gov.ezp1.harvard.edu/entrez/query.fcgi?holding=hulib&cmd=Retrieve&db=pubmed&dopt=Abstract&list_uids=16077717&query_hl=2); Int J Obes Relat Metab Disord. 2005 Aug 2; [↑](#endnote-ref-12)
12. [Nakanishi N](http://www.ncbi.nlm.nih.gov.ezp1.harvard.edu/entrez/query.fcgi?holding=hulib&db=pubmed&cmd=Search&term="Nakanishi+N"%5BAuthor%5D), [Shiraishi T](http://www.ncbi.nlm.nih.gov.ezp1.harvard.edu/entrez/query.fcgi?holding=hulib&db=pubmed&cmd=Search&term="Shiraishi+T"%5BAuthor%5D), [Wada M](http://www.ncbi.nlm.nih.gov.ezp1.harvard.edu/entrez/query.fcgi?holding=hulib&db=pubmed&cmd=Search&term="Wada+M"%5BAuthor%5D).Intern Med. 2005 Jun;44(6):542-7.) [↑](#endnote-ref-13)
